# “EVERYBODY HAS A SOUL”: PEOPLE WITH DEMENTIA AND NURSING STUDENTS CO-CREATING LIFE STORIES

**DOI:** 10.1093/geroni/igae098.1936

**Published:** 2024-12-31

**Authors:** Beth Mastel-Smith, Michelle Kimzey, Amy Ahlbrand Robinson, Melinda Hermanns

**Affiliations:** The University of Texas Tyler, Baker City, Oregon, United States; Texas Christian University, Fort Worth, Texas, United States; Freelance Artist, Missouri City, Texas, United States; The University of Texas Tyler, Tyler, Texas, United States

## Abstract

Lack of meaning and purpose in life can increase disability, stress, mortality, and the risk of cognitive impairment. Finding meaning and purpose helps people with dementia adjust to the diagnosis and can be accomplished via expressions of identity such as life review, a formal process of systematically reviewing and reflecting on one’s lived life. Digital storytelling involves telling a story using computer technology to record and share an individual’s narrative. The purpose of this study was to understand the perceived benefits of nursing students and people with dementia (the mentors) described after digitally co-creating the mentors’ life story. Allport’s Intergroup Contact Theory and the Lifestory as Legacy protocol guided intervention development. The study was approved by Institutional Review Boards and participants signed informed consent. Ten mentors and 13 students co-created the mentors’ life story during seven online meetings and completed qualitative interviews using an interview guide. Colaizzi’s process of data analysis was used to analyze data. Students and mentors described what it was like to co-create the mentor’s life story, what the experience meant to them, and what they learned. Mentors described how the student did and made them feel, importance of sharing their story with family, and advice for future health professionals regarding dementia care. Students commented on preparation to elicit their mentor’s stories and the perceived effect on their mentor. Co-creation of life stories online is feasible and had positive effects for nursing students and mentors with dementia.

